# Familial risk and protective factors in alcohol intoxicated adolescents: psychometric evaluation of the family domain of the Communities That Care Youth Survey (CTC) and a new short version of the Childhood Trauma Questionnaire (CTQ)

**DOI:** 10.1186/s12887-015-0471-z

**Published:** 2015-11-19

**Authors:** Heidi Kuttler, Hanna Schwendemann, Eva Maria Bitzer

**Affiliations:** Public Health & Health Education, Freiburg University of Education, Kunzenweg 21, 79117 Freiburg, Germany

## Abstract

**Background:**

Alcohol intoxicated adolescents (AIA) in emergency department are an important target group for prevention and valid information on their familial risk and protective factors (RPF) is crucial for implementing customized family-based counseling in hospitals. We therefore, examined the psychometric characteristics of scales which assess familial RPF.

**Methods:**

We used seven family scales from the Communities That Care Youth Survey Instrument (CTC-F7); four assess risk factors: family conflicts, poor family management, parental attitudes favorable towards drug use/antisocial behavior; three assess protective factors: family attachment, opportunities and rewards for prosocial involvement. To assess physical and emotional abuse and emotional neglect, we created a new scale composed of six items from the Childhood Trauma Questionnaire (CTQ-6). We tested these eight scales on 342 AIA aged 13-17. Based on the classical test theory we calculated descriptive item and scale statistics and internal consistency. We assessed construct validity by confirmatory factor analysis with Maximum Likelihood (ML) estimation in a sample with imputed missing values (EM-Algorithm). To check robustness, we repeated the analyses with complete cases, with multiple imputed data, and with methods suitable for categorical data. We used SPSS 21, AMOS 21 and R (randomForrest and lavaan package).

**Results:**

Three of seven CTC-F scales showed poor psychometric properties in the descriptive analysis. A ML-confirmatory model with five latent factors fitted the remaining CTC-F scales best (CTC-F5). The latent structure of the CTQ-6 is characterized by three first-order factors (physical abuse, emotional abuse, emotional neglect) and one second-order factor. The global goodness-of-fit indices for the CTC-F5 and the CTQ-6 demonstrated acceptable fit (for both models: TLI and CFI>0.97, RMSEA<0.05). The confirmatory evaluation based on complete cases (n=266), on multiple imputed data, and with alternative estimation methods produces global and local model-fit indices that are comparable to those from the main analysis. The final subscales CTC-F5 and CTQ-6 show acceptable to good internal consistency (α>0.7).

**Conclusions:**

The final CTC-F5 and the newly developed CTQ-6 demonstrate acceptable to good psychometric properties for the AIA sample. The CTC-F5 and the CTQ-6 facilitate a psychometrically sound assessment of familial RPF for this vulnerable and important target group for prevention.

## Background

One of the most significant risks worldwide for morbidity and mortality in young people is alcohol [1]. Excessive alcohol consumption in adolescence does not only point to future disorders but accompanied by other risk factors, it can be an indicator of already existing disorders or problems. The hospitalization of adolescents following acute alcohol intoxication presents a key opportunity for initiating preventive measures, and the sound measurement of the individual’s risks and resources are the basis for customized prevention. In Germany, prevention efforts for alcohol intoxicated adolescents (AIA) include support strategies for the entire family system [2]. A short but psychometric sound instrument to assess familial Risk and Protective Factors (RPF) could provide counseling practitioners with relevant information. In this paper, we present the psychometric evaluation of scales used to assess familial risk and protective factors among AIA.

### Excessive alcohol consumption as major health risk in adolescence

In Europe, 10 % of all deaths among young women are associated with alcohol consumption and at 25 % the death rate for men is even higher, namely 13,000 men between the age of 16 and 24 die annually from alcohol-related causes [3]. Early and excessive alcohol consumption is often linked to alcohol abuse later in life [1, 7–9] and to further behavioral problems [4–6]. Puberty is an especially vulnerable phase of life [10] and adolescents hospitalized due to alcohol intoxication are an at-risk group whose healthy development is threatened [11–14]. Family plays a critical role in fostering children’s positive development, and counseling of AIA has to take the whole family system into consideration. That is our motivation to evaluate measurements assessing RPF in the family. The implementation of timely early intervention measures based on the family’s risk profile could help ensure customized support measures and prevent mental health issues and negative developmental cascades among AIA.

### Familial risk and protective factors for adolescent development

Studies show that adolescents with substance abuse have less parental support and monitoring than their peers [15–17] and are more likely to grow up in families with parental addiction [18–20]. They are also frequently victims of sexual or physical abuse [21] which plays a central role in the development and persistence of many severe disorders and illnesses such as violent behavior [22], delinquency, depression [23] and other mental disorders [24, 25]. On the other hand, there is evidence that the buffering effect of protective factors increases with the increasing number of risk factors to which adolescents are exposed [26–29].

Models of risk and protective factors try to predict the onset and progression of disorders as a basis for planning effective preventive intervention [26, 27, 30–32]. The Social Development Model (SDM) provides a framework for explaining healthy or problematic development of adolescents. In this model, the family environment emerges as one of the main factors that influences adolescent development [4, 27, 28, 31, 33, 34]. In compliance with the SDM, protective familial factors are a) opportunities for adolescents’ positive involvement in the family b) promotion of such skills, and c) perceived rewards for prosocial behavior [35, 36]. Routine tasks and responsibilities within the family seem to be important protective factors especially for male adolescents [37]. Familial recognition for prosocial involvement has been identified as a protective factor for problem gambling in young adults [67]. Furthermore, an effect that could be seen across different cultures is that continuous parental monitoring protects against adolescent externalizing problem behavior [4]. Other significant protective factors are family attachment (conversations, outings), opportunities for prosocial involvement (confiding in parents in case of problems, active inclusion of adolescents), and recognition in the family (parents offer praise and are proud of their children) [27, 39]. Risk factors for a healthy development are low family attachment and weak parent–child bonding [40], lack of parental interest in children's school and friends, unclear and inconsistent rules, lack of parental control, severe family conflicts, and parental attitudes favorable towards antisocial behavior and substance abuse [27, 39].

The assessment of familial RPF could be the basis for counseling aimed at reducing family risk factors and amplifying protective factors. To our knowledge there is no established instrument for target groups with an elevated risk for developmental hazards (such as AIA), that assesses a broad array of familial RPF. With our study we want to take a first step in developing a validated instrument to measure family RPF, which can provide counselors in hospitals with the information needed to carry out customized prevention measures.

## Methods

### Study sample and study design

We conducted our study in the same setting as the instrument’s future application. Between June 2012, and October 2013 adolescents hospitalized following acute alcohol intoxication, aged 13 to 17 years, were surveyed in ten different hospitals throughout Germany [41]. The questionnaire-based survey was carried out at the patient’s bedside before the customary brief intervention measures of the alcohol prevention program “HaLT” [11, 42, 43]. Written consent of both, parents and adolescents, was collected by the specialized social workers together with the routine waiver of medical confidentiality for the HaLT-program, and sent to the study center in Loerrach (Germany). The questionnaire which was marked with a personal identification number was sent to the study center in Freiburg (Germany).

### Ethical approval

This study was approved by the ethic commission of the State Medical Association Baden-Wurttemberg, Germany (F-2012-035).

### Sample

The sample comprised 342 adolescents with an average age of 15.5 years (SD 1.21). 51.9 % were male. Seventeen percent of the candidates came from families with a migrant background. Less than half of the adolescents lived with both parents and 5.6 % were in institutional care (Table 1).

**Table 1:** Sociodemographic characteristics of the adolescents surveyed

	Number	in %
Age (years, Mean, SD)	308	15.5 (1.2)
Female sex	337	48.1
Family situation	342	
With biological parents		46.5
With mother only		23.1
With mother and her partner		16.1
In an institution		5.6
With father (and his partner)		5.5
Other		3.7
Migration background	336	17.0
Maternal employment status	327	
Full time		40.4
Part time		30.0
Not employed		19.6
Seeking employment		8.3
Other		1.7
Paternal employment status	299	
Full time		78.6
Part time		10.0
Not employed		5.7
Seeking employment		5.0
Other		0.7

### Instruments

#### Communities That Care Youth Survey – seven family subscales (CTC-F7)

The Communities That Care Youth Survey (CTC) developed within the US-American Communities That Care Network [27, 35, 44] contains a broad range of familial RPF. It was developed to establish measures for the prevention of substance abuse, delinquency, and other behavior problems among adolescents in communities [27, 39]. The CTC is based on the Social Development Model and has been used in the USA, Australia, the Netherlands, England, Scotland and Germany [17, 45]. A German version of the CTC with eight family scales was used in the Study to Addiction Prevention in Networks, “SPIN” [46]. Our CTC instrument contains seven family scales: family conflicts, poor family management, parental attitudes favorable towards drug use and parental attitudes favorable towards antisocial behavior, family attachment, opportunities for prosocial involvement and rewards for prosocial involvement (CTC-F7) (Table 2). The response categories range from 1 = “no” to 4 = “yes” or from 1 = “very wrong” to 4 = “very right”. The eighth scale pertaining to a family history of antisocial behavior (e.g. parental drug dealing or drug use, and prison experience) was not included in our test instrument because of the personal contact that the adolescents and the parents had with the interviewer, who was also the counselor in the prevention program.

**Table 2:** Initial risk and protective factor scales – family domain of the Communities That Care Youth Survey (CTC-F7)

Scale abbrev.	Family domain	Item abbrev.	Item description
FR_2	Poor family management	R45n	Parents ask about school performance
		R45a	Parents know where I am
		R45p	Parents notice when I come home late
		R45d	Parents want me to call if I am going to come home late
		R45g	Clear family rules
		R45e	Parent would notice if I use drugs
		R45f	Parents would find out if I skip school
FR_3	Family conflict	R45b	Frequent yelling in the family
		R45o	Repeated episodes of severe conflict
		R45c	Repeated yelling about the same things
FR_4	Parental attitudes favorable to drug use	R44b	Favorable attitude towards alcohol use
		R44d	Favorable attitude towards cigarettes
		R44e	Favorable attitude towards marijuana
FR_5	Parental attitudes favorable to antisocial behavior	R44a	Favorable attitude towards skipping school
		R44f	Favorable attitude towards stealing
		R44g	Favorable attitude towards antisocial behavior
		R44h	Favorable attitude towards child’s violent behavior
FP_1	Family attachment	P45h	Mother: feel close to
		P45j	Mother: communicate with
		P45k	Father: feel close to
		P45m	Father: communicate with
		P45i	Mother: enjoys spending time together
		P45l	Father: enjoys spending time together
FP_2	Family opportunities for prosocial involvement	P53e	Parents encourage family outings
		P53c	Parents actively include adolescents in decision making
		P53d	In case of problems can ask parents for help
FP_3	Rewards for prosocial family involvement	P53b	Parents offer praise
		P53a	Parents are proud

#### Creating a six-item short version of the Childhood Trauma Questionnaire

Family violence such as abuse and neglect are risks that could indicate the necessity of immediate professional intervention for AIA. The items in CTC-F do not cover this area. Therefore, we supplemented the CTC scales with items from the Childhood Trauma Questionnaire (CTQ). CTQ is a 28 item questionnaire, based on retrospective self-report and uses a five point Likert scale response system (1 = “never true” to 5 = “very often true”). It enjoys widespread international acceptance [48–51], has already been successfully tested on adolescents aged 12–17 years [47] and has been used in several German surveys [52–55]. The CTQ covers, among others, the domains (1) physical abuse, (2) emotional abuse, and (3) emotional neglect. We examined these three CTQ domains [53], looking for items with high factor loadings and high item-total correlation and selected the two items for each of the three domains which best matched both criteria (Table 3).

**Table 3:** The six-item short form from Childhood Trauma Questionnaire (CTQ-6)

Item	From the time of childhood until today …
R48d	I was hit with a belt, a stick or other hard object
R48c	People in my family hit me so hard it left bruises or marks
R48b	I thought my parents wished I had never been born
R48e	People in my family said hurtful or insulting things to me
R48ar	I felt loved
R48fr	People in my family felt close to each other

#### Psychometric evaluation

The psychometric evaluation of the CTC-family scales and the CTQ items was executed separately in multiple steps according to the classical test theory. First, we calculated descriptive item and scale statistics such as mean, proportion of missing values, item difficulty, item-total correlation, and internal consistency. Item difficulty was calculated using the mean value of one item of all subjects divided by the maximum value of this item. The item-total correlation is the correlation of one item with the scale, treating ordinal data as if they conform to interval scales. A Cronbach’s alpha higher than α = 0.8 is deemed as an adequate internal consistency for assessing interindividual differences [56, 57].

Secondly, we explored the uni-dimensionality of each of the initial scales with exploratory factor analysis (EFA) using the Maximum Likelihood method (ML). ML-EFA extracts factors step-by-step and assesses with a *χ*2 test whether the model fits the postulated structure across the entire population. The ML-EFA analyzes the shared variance of a variable to reveal the underlying factor structure [58].

Finally, construct validity was assessed by confirmatory factor analysis (CFA), which has been shown to be an adequate method for testing theoretically assumed factor structures of multidimensional scales. The ML method was used to estimate the parameters, a procedure suitable if a sufficient sample size is available. Modifications were made by using goodness-of-fit indices [59]. Indicator reliability (≥0.4), factor reliability (≥0.6), and average of measured variance (≥0.5) are measures used to assess the convergent validity of constructs at the local level [60, 61]. Usually a Chi-Square test is performed to evaluate models' global goodness-of-fit, but this test is not suitable for large samples such as ours. Therefore, we used the Comparative Fit Index (CFI), the Tucker Lewis Index (TLI), and the Root Mean Square Error of Approximation (RMSEA) to evaluate our models’ global goodness of fit. CFI and TLI values ≥ 0.95 and RMSEA ≤ 0.05 indicate good model fit [61].

The main analyses were carried out with a sample that had missing values imputed by the Expectation Maximization (EM) Algorithm. EM is an effective, but not perfect technique to manage missing data. As a sort of sensitivity analysis we repeated the CFA (1) on the complete cases and (2) with multiple imputations (*N* = 1000), to assure that the use of single imputation did not produce parameter estimates highly dependent on the imputed values [62]. Because of the non-normal distribution and categorical type of data we performed the analysis using the bootstrapping ML method and we calculated the approximate model fit value Standardized Root Mean square Residual (SRMR) (≥0.10) [63]. Furthermore, we used polychloric correlation matrices as input for CFA and Diagonally Weighted Least Squares (DWLS) and robust measures for non-normal distributed categorical data estimation methods [64, 65]. Weighted Least Square Mean-Variance (WLSMV) adjusted estimators were used to obtain appropriate fit indices. Additionally, we computed the Weighted Root Mean Square Residual (WRMR) as an approximate model fit value.

The descriptive analysis, the internal consistency analysis, EM imputation, and EFA were calculated with SPSS Version 21.0. The CFA using the ML was performed with AMOS software 21.0. Multiple imputed data sets were created with the randomForest package of R. For the additional CFA we used the lavaan (0.5.-18) package for structural equation modeling implemented in the R system for statistical computing [66].

## Results

### Descriptive item and CTC-F7 subscales and CTQ-6 characteristics

The descriptive statistics for all initial scales, based on the original sample without imputed missing values are summarized in (Table 4). The missing data in the sub-scales of CTC-F7 and CTQ-6 vary between 4.7 and 12.3 %. Scales with more items show a higher proportion of missing data. Item difficulty and item-total correlation show a high degree of heterogeneity. The CTC-FR_4 subscale “parental attitudes favorable to drug use” and CTC-FR_5 subscale “parental attitudes favorable to antisocial behavior” do not perform well. The item-total correlation is low (r_itc_ between 0.25 and 0.45) and the item difficulty is high (p_i_ between 0.25 and 0.33). Four of the seven CTC-F7 subscales and the CTQ-6 reveal a satisfactory to acceptable internal consistency. The two scales “parental attitudes favorable to drug use” (FR_4) and “parental attitudes favorable to antisocial behavior” (FR_5) show low internal consistency, as does the FR_2 scale “poor family management” (Table 4).

**Table 4:** Initial CTC-F7 and CTQ-6 – descriptive item and scale values

Domain abbrev.	Domain	N items	Missing %	M (Max)	Cα	ritc Min-Max	Pi	EFA Min-Max
FR_2	Poor family management	7	9.1	22.7 (28)	0.69	0.32 – 0.47	0.72 – 0.86	0.4 – 0.59
FR_3	Family conflict	3	7.9	6.2 (12)	0.81	0.60 – 0.74	0.44 – 0.57	0.66 – 0.90
FR_4	Parental attitudes favorable to drug use	3	6.1	3.8 (12)	0.40	0.25 – 0.30	0.25 – 0.33	0.39 – 0.53
FR_5	Parental attitudes favorable to antisocial behavior	4	4.7	4.5 (16)	0.56	0.25 – 0.45	0.25 – 0.29	0.37 – 0.65
FP_1	Family attachment	6	12.3	17.2 (24)	0.79	0.47 – 0.67	0.51 – 0.79	0.37 – 0.93
FP_2	Family opportunities for prosocial involvement	3	8.2	9.4 (12)	0.74	0.53 – 0.60	0.68 – 0.76	0.63 – 0.79
FP_3	Rewards for prosocial family involvement	2	6.7	6.5 (8)	0.87	0.77	0.74 – 0.78	-
CTQ-6	Physical abuse, emotional abuse, emotional neglect	6	10.5	4.6 (24)	0.82	0.49 – 0.80	0.25 – 0.41	0.57 – 0.79

CTC = Communities that Care Youth Survey Instrument; CTQ = Childhood Trauma Questionnaire; M = Mean Value, Cα = Cronbach’s α total scale, r_itc_ = Item-Total Correlation, p_i_ = Item Difficulty, EFA = Factor Weighting in Exploratory Factor Analysis

### Exploratory assessment of uni-dimensionality of CTC-F7 subscales and CTQ-6

The EFA results are based on the single EM imputed data. EFA produced satisfactory one-factor models only with the FR_5 scale “parental attitudes favorable to antisocial behavior” and the CTQ-6. The other scales had either insufficient model fits or were underidentified. For example, for the FR_2 scale “poor family management”, the *χ*^2^ test of model fit is significant *χ*^2^ (14) = 46.39; *p* < 0.00. This indicates that the model is not well defined. Furthermore, the CTC subscale FR_4 “parental attitudes favorable to drug use” shows negative degrees of freedom in the EFA. This also points to an underidentified model. The *χ*^2^ test for a one-factor solution is also significant (*χ*^2^ (9) = 33.06; *p* < 0.00) for the FP_1 scale “family attachment” which refers to both parents. Relaxing EFA-model constraints and allowing for factors with an Eigen value larger than one result in a two-factor solution that distinguishes items concerning the mother from those concerning the father.

In summary, the evaluation of the descriptive item statistics, internal consistency, and the exploratory analysis of construct validity exhibit obvious deficiencies for four of seven scales.

### Confirmatory factor analysis – part 1: from CTC-F7 to CTC-F5

The results presented here are those from the main analysis, which means single EM imputed data and ML-CFA. The initial analysis included all 28 items of CTC-F7 and aimed to replicate the seven first order latent factors. However, this CFA-Model does not display satisfactory model fit, row “CTC-F7 initial” (Table 5).

**Table 5:** Initial and final CTC-F7 and CTQ-6 - confirmatory factor analysis (ML method, EM imputation; global goodness-of-fit indices)

Model**/**Fit index	*Χ* ^2^	df	*Χ* ^2^/ df	p	TLI	CFI	RMSEA
Acceptable Fit			<3		>0.95	>0.95	<0.08
Good Fit			<2	>0.05	>0.97	>0.97	<0.05
CTC-F7 initial	1193.93	329	3.63	0.00	0.72	0.75	0.088
CTC-F5 final	91.14	62	1.47	0.009	0.98	0.99	0.037
CTQ-6 initial	193.86	9	21.54	0.00	0.61	0.76	0.25
CTQ-6 final	15.08	6	2.51	0.02	0.97	0.99	0.07

CTC = Communities that Care Youth Survey Instrument; CTQ = Childhood Trauma Questionnaire; *Χ* ^2^ = Chi-Squared; df = degrees of freedom; *Χ* ^2^/df = Standardized Chi-Squared; TLI = Tucker-Lewis Index; CFI = Comparative Fit Index; RMSEA = Root Mean Square Error of Approximation

Results of the additional analyses are summarized in Table 8, Table 9, Table 10 and Table 11 and referred to where appropriate.

**Fig. 1:**
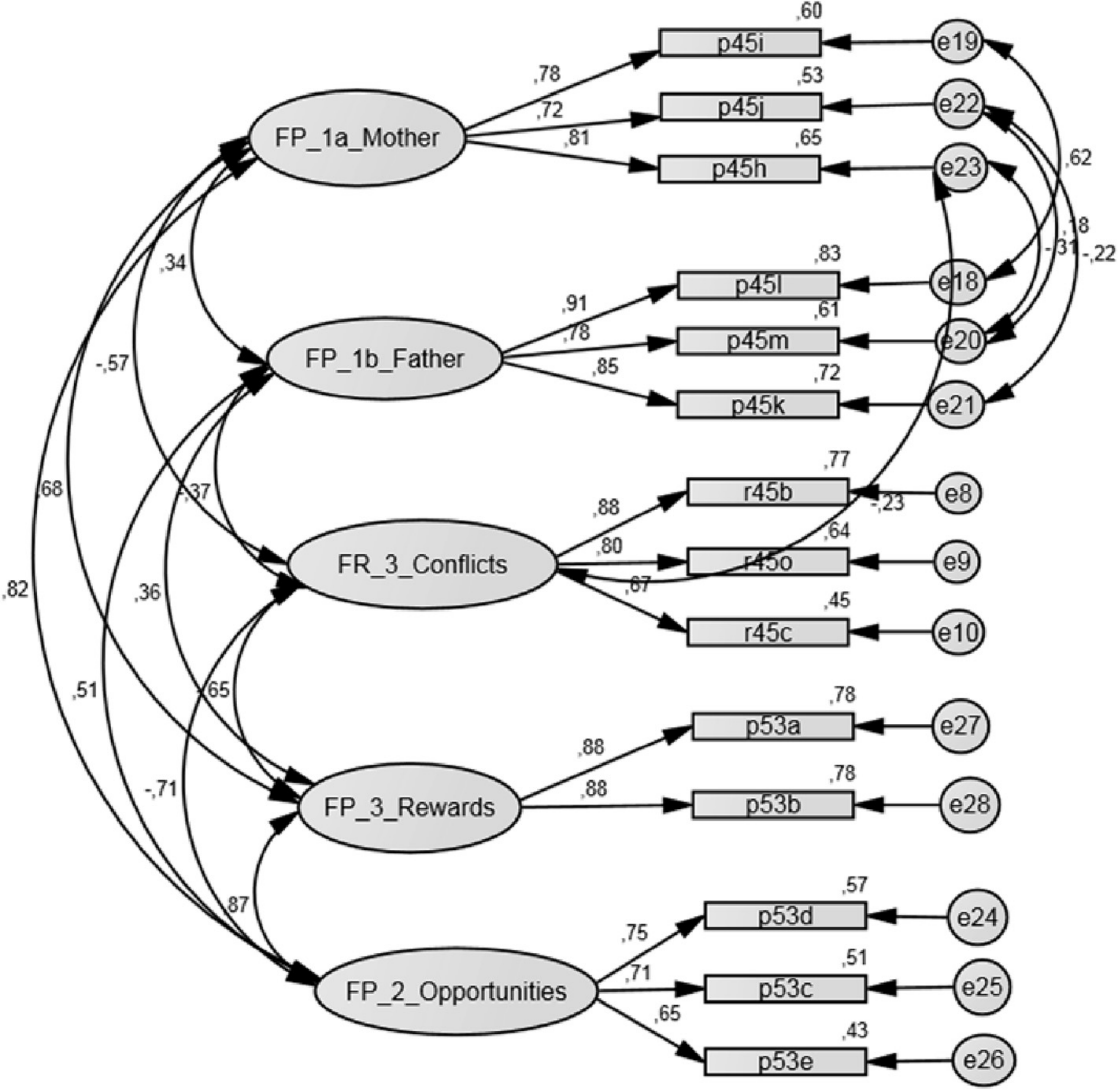
Final structural equation model – CTC-F5

The descriptive item analysis, the CFA process and the evaluation of global goodness-of-fit indices led to the elimination of three scales: FR_2 “poor family management”, FR_4 “parental attitudes favorable to drug use”, and FR_5 “parental attitudes favorable to antisocial behavior”. Based on the EFA and the residual correlations which point to its two-dimensional structure the FP_1 scale “family attachment” was divided into two scales: FP_1a “attachment to mother” and FP_1b “attachment to father”. The division leads to an improvement in the model, but only when strong correlations of the error terms between the (now) two scales are permitted. Also, the residual correlation between the construct “family conflict” (FR_3) and the item p45h (Do you get along with your mother?) (r = 0.23) points to difficulties. Estimating the CTC-F5 model separately in subgroups of adolescents living either (a) with both parents, (b) with a single mother and new partner or (c) in another family situation (e.g. juvenile shelter, living alone) shows: the residual correlations between FP_1a “attachment to mother” and FP_1b “attachment to father” are much lower in models b and c than in model a. Indicators of the latent construct “parental/mother/father attachment” may not measure the same construct in adolescent groups differing by family structure. A formal assessment of measurement invariance was beyond the scope of this analysis and for the time being we think the two factor solution is more appropriate than the single factor solution, because a substantial proportion of the adolescents live in single parent families. The final structure of the (modified) CTC-F5 is displayed in Fig. 1.

The local model fit indices of the final CTC-F5 model range with regard to the values of the standardized factor weighting between 0.65 and 0.91 and indicator reliability is always >0.4 (Table 6). Item p53e (My parents frequently want me to do things together with them) has the lowest weighting within the FP_2 scale “opportunities for prosocial involvement”. There is a correlation of r = 0.82 between the construct “mother” and the FP_2 scale. There is further correlation between “mother” and the FP_3 scale “rewards for prosocial involvement” (r = 0.68) and between the two constructs FP_2 and FP_3 (r = 0.87). There is a negative correlation between FP_1a “mother” and FR_3 “family conflict” (r = −0.57), between FR_3 and FP_2 (r = −0.71), as well as FR_3 and FP_3 (r = −0.65) (Fig. 1).

**Table 6:** Final CTC-F5 and CTQ-6 - local goodness-of-fit criteria (ML method, EM imputation)

Scale abbrev.	Item abbrev.	Indicator-reliability	Weight	t-Value of factor weight	Factor-reliability	AVE
Acceptable fit indices		≥0.4	≥0.5		≥0.6	≥0.5
FR_3					0.82	0.61
	R45b	0.77	0.88	1^a^		
	R45o	0.64	0.80	16.1***		
	R45c	0.45	0.67	13.06***		
FP_1a					0.81	0.58
	P45h	0.65	0.81	1^a^		
	P45j	0.53	0.72	14.19***		
	P45i	0.60	0.78	14.51***		
FP_1b					0.89	0.72
	P45k	0.72	0.85	19.93***		
	P45m	0.61	0.78	18.18***		
	P45l	0.83	0.91	1^a^		
FP_2					0.75	0.50
	P53e	0.43	0.65	11.76***		
	P53c	0.51	0.71	12.84***		
	P53d	0.57	0.75	1^a^		
FP_3					0.87	0.78
	P53b	0.78	0.88	19.38***		
	P53a	0.78	0.88	1^a^		
CTQ-6					0.90	0.60
	R48ar	0.79	0.89	10.24***		
	R48fr	0.48	0.69	8.05***		
	R48b	0.57	0.76	1^a^		
	R48e	0.58	0.76	11.54***		
	R48d	0.52	0.72	1.00***		
	R48c	0.94	0.97	10.99***		

CTC = Communities That Care Youth Survey Instrument; CTQ = Childhood Trauma Questionnaire

*** *p* ≤ 0.001; AVE = Average Variance Extracted; ^a^ = parameter fixed to the value 1 to allow identification

Indices of global goodness of fit of the CTC-F5 are summarized in Table 5. The modified CTC-F5 model is improved in comparison with the initial model and shows good to acceptable global and local fit. All values are within an acceptable range and the modified models also display satisfactory local values.

The final model for the CTC-family domain consists of five subscales: the risk-factor scale: FP_3 “family conflict” and the protective-factor scales: FP_1a attachment to mother, FP_1b attachment to father, FP_2 “opportunities for prosocial involvement” and FP_3 “rewards for prosocial involvement”. The descriptive statistics of the modified CTC-F5 subscales also show satisfactory results (Table 7).

**Table 7:** Final CTC-F5 and CTQ-6 - descriptive item und subscale values

Scale abbrev.	Family domain	N items	Missing %	M (Max)	Cα	ritc Min-Max	Pi	EFA Min-Max
FR_3	Family conflict	3	7.9	6.2 (12)	0.81	0.60 – 0.74	0.44 – 0.57	0.66 – 0.90
FP_1a	Attachment to mother	3	8.2	9.1 (12)	0.80	0.64 – 0.66	0.63 – 0.69	0.75 – 0.78
FP_1b	Attachment to father	3	9.9	8.1 (12)	0.88	0.71 – 0.81	0.51 – 0.70	0.75 – 0.78
FP_2	Family opportunities for prosocial involvement	3	8.2	9.4 (12)	0.74	0.53 – 0.60	0.68 – 0.76	0.63 – 0.79
FP_3	Rewards for prosocial family involvement	2	6.7	6.5 (8)	0.87	0.77	0.74 – 0.78	-
CTQ-6	Physical and emotional abuse and emotional neglect	6	10.5	4.6 (24)	0.82	0.49 – 0.80	0.25 – 0.41	0.57 – 0.79

CTC = Communities that Care Youth Survey Instrument; CTQ = Childhood Trauma Questionnaire; M = mean value, Cα = Cronbach’s total scale, r_itc_ = item total correlation, p_i_ = item difficulty, EFA = factor loading in Exploratory Factor Analysis

To check if the results were biased because of the non-optimal estimation method, we performed (1) a CFA using the complete cases (*n* = 266, results not presented). This leads to model-fit values comparable to those with imputed data (*n* = 342). (2) We also analyzed the model using multiple imputed data (*N* = 1000). The results presented in Tables 8, 9 and 10, return good model-fit values.

**Table 8:** Initial and final CTC-F7 and CTQ-6 - confirmatory factor analysis (multiple imputation and bootstrapping ML, global goodness-of-fit indices)

Model/Fit indices	*Χ* ^2^	df	*Χ* ^2^/ df	p	TLI	CFI	RMSEA	SRMR
Acceptable Fit			<3		>0.95	>0.95	<0.08	
Good Fit			<2	>0.05	>0.97	>0.97	<0.05	≤0.10
CTC-F7 initial	11796.92	329	358.4	0.00	0.65	0.70	0.10	0.11
CTC-F5 final	9301.71	62	150.03	0.00	0.95	0.97	0.07	0.03
CTQ-6 initial	20669.61	9	2296.62	0.00	0.58	0.75	0.26	0.10
CTQ-6 final	1581.29	6	263.55	0.00	0.95	0.98	0.09	0.03

CTC = Communities that Care Youth Survey Instrument; CTQ = Childhood Trauma Questionnaire; *Χ* ^2^ = Chi-Squared; df = degrees of freedom; *Χ* ^2^/df = Standardized Chi-Squared; TLI = Tucker-Lewis Index; CFI = Comparative Fit Index; RMSEA = Root Mean Square Error of Approximation

**Table 9:** Final CTC-F5 and CTQ-6 - confirmatory factor analysis (multiple imputation and bootstrapping ML, local goodness-of-fit criteria)

Scale abbrev.	Item abbrev.	Indicator-reliability	Weight	t-Value of factor weight	Factor-reliability	AVE
Acceptable Fit		≥0.4	≥0.5		≥0.6	≥0.5
FR_3					0.82	0.61
	R45b	0.77	0.87	1^a^		
	R45o	0.62	0.71	158.0***		
	R45c	0.44	0.67	129.41***		
FP_1a					0.80	0.58
	P45h	0.64	0.80	1^a^		
	P45j	0.53	0.73	140.62***		
	P45i	0.59	0.77	141.47***		
FP_1b					0.88	0.71
	P45k	0.71	0.84	191.07***		
	P45m	0.58	0.76	172.04***		
	P45l	0.83	0.91	1^a^		
FP_2					0.75	0.50
	P53e	0.43	0.66	116,12***		
	P53c	0.51	0.72	126.92***		
	P53d	0.55	0.74	1^a^		
FP_3					0.87	0.77
	P53b	0.77	0.88	187.49***		
	P53a	0.77	0.88	1^a^		
CTQ-6					0.89	0.59
	R48ar	0.84	0.92	101.923***		
	R48b	0.55	0.74	1^a^		
	R48e	0.60	0.77	107.43***		
	R48fr	0.48	0.70	83.17***		
	R48c	0.93	0.96	109.24***		
	R48d	0.52	0.72	94.8***		

CTC = Communities that Care Youth Survey Instrument; CTQ = Childhood Trauma Questionnaire; *** *p* ≤ 0.001; AVE = Average Variance Extracted; ^a^ = parameter fixed to the value 1 to allow identification

**Table 10:** Final CTC-F5 and CTQ 6 - bootstrapping estimates of standard error

Scales	Item abbrev.	SE	SE-SE	Mean	Bias	SE-Bias
CTC						
FR_3	R45b	0.002	0.00	0.875	0.00	0.00
	R45o	0.004	0.00	0.790	0.00	0.00
	R45c	0.003	0.00	0.667	0.00	0.00
FP1b	P45l	0.002	0.00	0.910	0.00	0.00
	P45m	0.002	0.00	0.763	0.00	0.00
	P45k	0.003	0.00	0.841	0.00	0.00
FP2	P53d	0.004	0.00	0.745	0.00	0.00
	P53c	0.004	0.00	0.717	0.00	0.00
	P53e	0.004	0.00	0.658	0.00	0.00
FP3	P53a	0.004	0.00	0.878	0.00	0.00
	P53b	0.003	0.00	0.880	0.00	0.00
FP1a	P45h	0.003	0.00	0.802	0.00	0.00
	P45j	0.004	0.00	0.729	0.00	0.00
	P45i	0.004	0.00	0.767	0.00	0.00
CTQ-6	Emotional_neglect	0.00	0.00	1.00	0.00	0.00
	Emotional_abuse	0.021	0.001	1.099	0.001	0.001
	Physical_abuse	0.015	0.001	0.621	0.001	0.001
Emotional_neglect	R48ar	0.00	0.00	1.0	0.00	0.00
	R48fr	0.01	0.001	0.784	0.001	0.001
Emotional_abuse	R48b	0.00	1.00	0.00	0.00	0.00
	R48e	0.018	0.001	1.3	0.001	0.001
Physical_abuse	R48c	0.021	0.001	1.452	0.00	0.001
	R48d	0.00	1.00	0.00	0.00	0.00

CTC-F5 = Communities that Care Youth Survey Instrument, family scales; CTQ-6: Six item short form of the Childhood Trauma Questionnaire; SE: Standard Error

This shows that it is unlikely that substantial distortion is caused by single imputation of the missing values. The CFA with bootstrapping method shows that the standard errors are not biased (Table 10). CFA with multiple imputed data, polychoric correlations as input and robust estimation methods for categorical data leads to comparable results presented here (Table 11).

**Table 11:** Initial and final CTC-F5 - confirmatory factor analysis (polychoric correlation matrix as CFA input, diagonally weighted least squares estimation & robust methods)

Model**/**Fit indices	*Χ* ^2^	df	*Χ* ^2^/ df	p	TLI	CFI	RMSEA	WRMR
Acceptable Fit			<3		>0.95	>0.95	<0.08	
Good Fit			<2	>0.05	>0.97	>0.97	<0.05	
CTC-F5 DWLS Model A	14967.4	91		0.00	1	1	0.02	0.4
CTC-F5 Robust Model A	5394:86	91		0.00	0.98	0.99	0.06	0.4
CTC-F5 Robust Model B	5394:86	91		0.00	0.99	0.99	0.05	0.34

CTC = Communities that Care Youth Survey Instrument; DWLS = Diagonally Weighted Least Squares, Robust; *Χ* ^2^ = Chi-Squared; df = degrees of freedom; *Χ* ^2^/df = Standardized Chi-Squared; TLI = Tucker-Lewis Index; CFI = Comparative Fit Index; RMSEA = Root Mean Square Error of Approximation; WRMR = Weighted Root Mean Square Residual

Model A: without correlation between latent variable FR_3_Conflict and the measurement error of item p45h (e23)

Model B: with correlation between latent variable FR_3_Conflict and the measurement error of item p45h (e23)

### Confirmatory factor analysis – part 2: CTQ-6

**Fig. 2:**
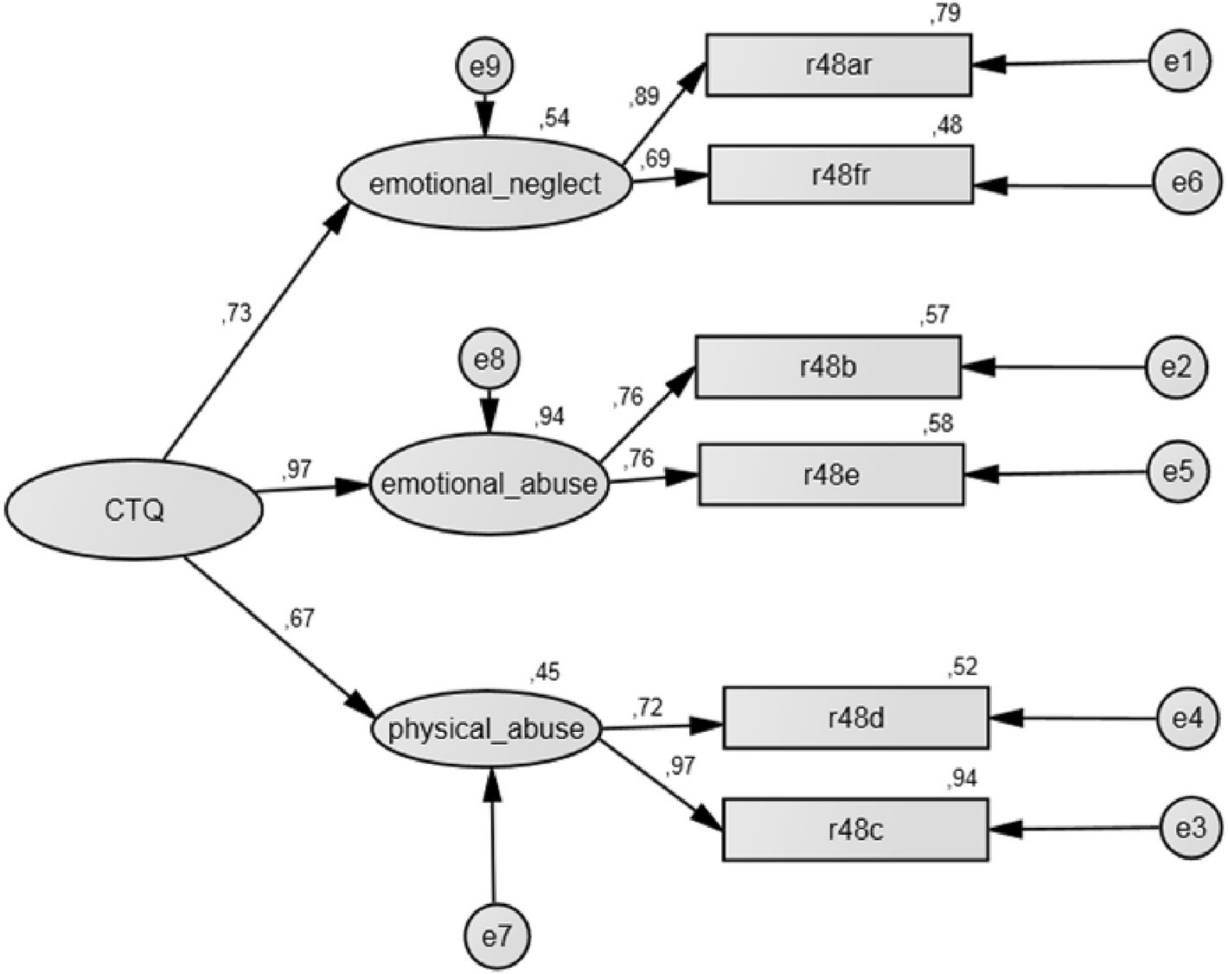
Final structural equation model – CTQ-6

The initial ML-CFA with EM imputed data of the six-item short version of the CTQ with one first order factor does not fit the data well (Table 5, row “CTQ-6 initial”). Based on the modification indices [59] which indicated a reduction of the *χ*^2^ statistics, a model where the two items of each dimension were explained by a latent first-order factor each, and a general second-order factor explaining the three first-order factors (physical abuse, emotional abuse and emotional neglect) fitted the data well (Fig. 2). With this structure, the final model displays very good local and global goodness-of-fit (Tables 5, and 6).

The CFA based on complete cases (*n* = 266, results not presented) and based on multiple imputed data sets (*N* = 1000) (Tables 8, 9, and 10) produces model-fit values comparable to those from the analysis with imputed data (*n* = 342). This also prevents bias caused by imputation. The underlying structure of the newly derived CTQ-6 short version is similar to that of the original long version, indicating construct validity.

## Discussion

It was our objective to conduct a psychometric evaluation and optimization of a collection of scales which assess familial RPF in individuals who belong to a vulnerable group i.e. young alcohol intoxicated patients. We combined seven CTC scales to assess familial RPF for adolescents. Originally, these scales were used to differentiate between groups with specific risk profiles as a reference for community prevention planning. Because the CTC-F7 scales do not assess physical and emotional abuse and emotional neglect - severe threats to the healthy development of AIA which could require intense or immediate professional intervention – we designed a CTQ brief scale with six items, two from each of the domains mentioned above.

Descriptive, exploratory and confirmatory analysis revealed that three of the seven CTC-F7-scales show poor psychometric properties in AIA. Those three CTC-family subscales are “poor family management” and especially “parental attitudes favorable to drug use” (α = 0.40) and “parental attitudes favorable to antisocial behavior” (α = 0.56). The authors of the original instrument which has been tested in the United States report that the internal consistency of the CTC-family subscale ranges from 0.62 to 0.83 [27]. In an Australian school survey [38], the internal consistency of the family-RPF scale ranges from α = 0.72 to 0.81. Due to the fact that the three scales mentioned above also performed rather poorly in the German SPIN study of school children with values of α = 0.59 (parents' attitudes favorable to drug use) and α = 0.70 (parents' attitudes favorable to antisocial behavior) [29] (personal communication), we think the better performance within the USA and Australian surveys is not only due to the very different target group surveyed in the samples (AIA vs. school children), but can be partly explained by the difference of parenting styles between Germans, U.S. Americans and Australians.

A factor contributing to the particularly low internal consistency of the CTC-subscales “parental attitudes favorable to drug use” and “parental attitudes favorable to antisocial behavior” in our survey might be the setting. In the German SPIN survey, the internal consistency of these scales was lower than it was in the US and Australian surveys but higher than in ours. It seems plausible that the overwhelming majority of adolescents hospitalized for alcohol intoxication felt that their parents would not accept drug use and antisocial behavior and answered these items more uniformly because their alcohol-related hospitalization had probably caused conflict with their parents. In summary, we would not recommend the use of these three scales in AIA due to their unsatisfactory psychometric properties.

The confirmatory factor analysis of the CTC-F5 not only portrays an adolescent’s close relationship to both parents plausibly, but also shows significant differences between the family roles of the mother and the father within the different samples in Germany and the United States. In our sample, a relatively high negative correlation can be detected between the mother and “family conflict” (r = −0.57). In the US study, there was low negative correlation between both parents and the “family conflict” subscale (r = −0.25) [44]. In the AIA sample mothers offer adolescents more “opportunities for prosocial involvement” than fathers do (r = 0.82/r = 0.51) and show more “rewards for prosocial involvement” (r = 0.68/r = 0.36). In the US study we find a higher correlation for fathers with regard to prosocial involvement than in our German study: “opportunities for prosocial involvement” (r = 0.63) and “rewards for prosocial involvement” (r = 0.51) [44]. Mothers in the German sample play a much more influential role in the children’s upbringing than fathers do. This difference is less pronounced in the US sample.

Our final CTC-F5, with two scales created by the division of the family attachment scale provides satisfactory model fit and a plausible latent structure. In a CTC survey conducted in the USA, the postulated model also could not be corroborated with regard to the scale “family attachment” and, like ours, it was divided into two constructs “attachment to mother” and “attachment to father”. This generated a model that described the data well and had a satisfactory model-fit index (*χ*^2^(629) = 120.19; TLI = 0.97; RMSEA = 0.06) [44]. The latent construct “family attachment” entails further investigation because our data indicate that adolescents living with both parents might conceptualize it differently than those living with a single parent. A formal assessment of measurement invariance for these scales should be carried out in a next step.

Though Glaser emphasizes the fact that the CTC Survey was not created as a diagnostic instrument for individual comparisons but as a tool for planning community prevention strategies [44], the psychometric properties of the CTC-F5 scales presented here warrant their use to describe individual risk profiles for adolescents hospitalized for acute alcohol intoxication.

### CTQ-6

The original three CTQ subscales emotional and physical abuse and emotional neglect showed satisfactory internal consistency in a German representative sample (physical abuse α = 0.89; emotional abuse α = 0.80; emotional neglect α = 0.83) [53]. Our abridged six-item ultra-short version not only replicates the original three factorial structure but also conforms to a general (second order) factor that could be called “childhood abuse and neglect”. In our AIA sample, it has an internal consistency sufficiently high to be used for individual comparisons. We think the CTQ-6 is a very promising short tool to assess childhood abuse and neglect under time constraints in preventive or clinical practice and its use in further applications like the screening of AIA merits further research.

### Limitations

One limitation to our findings is caused by the organizational structure of the survey which was carried out within the context of the prevention program HaLT by specialized social workers. Our test conditions optimally mirror the future setting of the planned instrument’s implementation. However, the personal contact with prevention personal might have caused bias towards social desirability.

Additionally, the results on the construct validity are limited by the fact that the final models are based on a fitting process in a single sample. Our attempts to check for the robustness of the main analyses cannot overcome this problem, but the high congruence of these results is promising. However, to be sure that the models are generalizable and not over-fitted to the current dataset, replication in an independent sample is required.

A further point is the measurement equivalence of the CTC-F5 and the CTQ-6. As we mentioned, some of the family scales seem to have different latent structures depending on the adolescent’s family structure. This should be investigated in further analyses, maybe with other samples e.g. samples from the CTC survey or the SPIN survey. Other aspects of measurement invariance concern the extent to which the psychometric properties of the CTC-F5 and the CTQ-6 are transportable or generalizable across other groups (e.g. gender, ethnicity). Glaser verified the applicability of the CTC survey in respect to differences in ethnicity and sex [44]. In our case, a comparable analysis is also called for since 17 % adolescents come from families with a migrant background. Unfortunately, this is not possible because our sample is too small.

Last, our results are just a snap shot and cannot verify the predictive ability of the tool. Though, the predictive ability of the CTC survey instrument has been assessed within the framework of the International Youth Development Study (IYDS) on problem gambling [67] and in studies on alcohol and substance abuse in adolescence [68]. In our case, the valid measure of the key familial RPF and developmental hazards using two abridged tools was developed for a special group of adolescents at risk of abusing alcohol.

It would be beneficial if the implementation of this tool could be tested in other subpopulations with an elevated risk for developmental hazards, for example, adolescents in residential or non-residential youth care services.

## Conclusion

In combination, CTC-F5 and CTQ-6, two brief, internally consistent instruments with promising construct validity, create an effective tool to assess familial risk and protective factors as well as childhood abuse and neglect in an already vulnerable group of adolescents, i.e. those hospitalized following acute alcohol intoxication. The tool’s psychometric characteristics warrant its implementation in customized preventive services for adolescents and their families. However, these findings require replication in an independent sample.
